# Supplementary material for: Understanding university students’ experiences of sitting while studying at home: A qualitative study
Source: PLoS One. 2024 Dec 6;19(12):e0314768. doi: 10.1371/journal.pone.0314768 (PMC11623464; doi:10.1371/journal.pone.0314768)
Supplement: S1 File — (DOCX) [file pone.0314768.s001.docx]

**Supplementary File: Interview Guide**

**Opening questions**

1. Before we begin, can you tell me a bit about what year you are in and what you study?
2. What does your weekly timetable look like?
3. How many hours are you on campus versus studying from home?
4. (If applicable) How did you get on with studying from home during the last academic year, during the Covid-19 pandemic?

**Studying at home - practices and priorities**

University-level studying involves doing some learning activities on campus – for example, attending seminars, as well as some at-home activities such as reading, watching lecture videos, and so on. But I’m interested in knowing how you go about studying *at home*, and why you study in the way that you do.

1. Focusing now on your experience of studying from home this year, can you describe a typical at-home study session for you?
   1. *Possible prompt:* Can you describe your typical home learning environment?
   2. *Possible prompt*: Are you able to study for as long as you’d like or are there ever interruptions to your study?
2. Now let's talk about your routines when studying from home. Are there things you do every time you study from home?
3. *Possible prompt:* What sort of things help you focus?
4. *Possible prompt:* Do you follow a particular schedule? Tell me more about that/your approach?

**NOTE TO RESEARCHER, only ask if not yet covered by participant**:

1. Do you feel your studying patterns have changed because of the coronavirus pandemic?
2. What works well for you when you study from home?
   1. *Possible prompt:* Anything else?
   2. *Possible prompt:* How does this compare when working elsewhere e.g., university, library etc?
   3. *Possible prompt:* How would you define a 'good day' regarding studying from home?
      1. Why is XXX [use language student uses e.g., being productive] important to you?
3. What doesn’t work so well for you when studying from home?
   1. What factors create a good day in comparison to a not so good day when working from home? (Note: could use language here that student uses in previous question e.g., ‘what factors create a productive day in comparison to an unproductive day?’
      1. How do you manage/control these factors? If you have been unable to manage such factors, what do you think would help you manage them?
4. What would improve your experience of studying from home?
   1. Anything else?

**Sitting time:**

We're interested specifically in how long you spend sitting while studying.

1. How much time would you say you spend sitting during a typical session of studying at home?
2. Do you ever think about how long you've been sitting while you're studying?
   1. *Possible prompt:* Do you monitor how long you've been sitting? (How?)
3. Can you tell me what you know about current health advice regarding sitting for long periods?
4. What do you think are the potential consequences of sitting for long periods?

**Data-driven questions:**

Now, I’d like to walk you through some responses you gave to the questionnaire ...

1. When asked how many breaks from sitting you take during an hour of sitting while studying, you answered X… Can you explain this in more detail for me?
   1. *Possible follow-up*: What determines whether and when you take breaks from sitting?
   2. *Possible follow-up*: How long are these breaks from sitting and where are they?
   3. *Possible follow-up*: What, if anything, might extend one of your breaks from sitting?
2. When asked the total estimated time you spend in short physical activity breaks during a typical study session at home you said ...  Can you explain this in more detail for me?
3. *Possible follow-up:* Can you describe what these short physical activity breaks involve?
4. *Possible follow-up:* What determines whether or not you take these breaks?
5. We asked you about whether you had psychological capability – in other words, the knowledge, skills, or headspace – to break up and limit your sitting time, you responded X ...  Can you explain this in more detail for me?
   1. *Possible follow-up*: What are the psychological barriers preventing you from breaking up/limiting your sitting time? Have you ever tried to overcome these?
6. We asked you about whether you had the physical opportunities to break up and limit your sitting time, and you responded X ...  Can you explain this in more detail for me?
   1. *Possible follow-up*: What aspects of your physical environment at home influence your opportunities to break up/limit your sitting time?
7. When asked whether you believe you have social opportunities to break up/limit your sitting time, you responded ‘X’. Can you explain this in more detail for me?
8. *Possible follow-up:* When do you feel like you have these social opportunities?
9. *Possible follow-up:* Where do these social opportunities usually occur?
10. When asked whether you are motivated to break up and limit your sitting time, you responded ‘X’. Can you explain this in more detail for me?
    1. *Possible follow-up:* What motivates you to break up your sitting time while studying?

**Understanding sitting attitudes: hypothetical scenarios and intervention ideas**

1. What would help you personally to sit less while studying at home?
2. What would help you personally to take more breaks from sitting while studying at home?

***NOTE TO RESEARCHER:* Participants may well feel they have answered these questions in the previous section, in which case you may not need to ask them again.**

1. Have you heard of anything that helps people to break up their sitting time and take more breaks?

*Possible prompts:*

- 1. Standing desks - What do you think about the idea of using a standing desk, as a way of reducing sitting? Would you consider using one at home?
  2. Pedal devices - these are devices you put under your desk and pedal while you're sitting down. They are a way of keeping you active even when you're sitting.  Would you consider using one at home?
  3. Phone/apps - you can use your phone to give you reminders to take breaks. Would you consider using that?

**The main aim of this study is to understand sitting and studying practices so that, in the future, we can develop strategies to encourage students to sit less when studying at home.**

1. If *you* were in our position, and *you* had to encourage university students to sit less when studying at home, what strategies would you propose doing?
2. What do you think might help you to improve your overall health when working from home?

**In addition, the research team are aiming to develop a resource to support students studying from home.**

1. Do you have any suggestions for what should be included in this resource?
2. What are the most important factors to focus on to support studying from home, and why?
