# Supplementary material for: Understanding university students’ experiences of sitting while studying at home: A qualitative study
Source: PLoS One. 2024 Dec 6;19(12):e0314768. doi: 10.1371/journal.pone.0314768 (PMC11623464; doi:10.1371/journal.pone.0314768)
Supplement: Attachment — Submitted filename: Response to Reviewers.docx [file pone.0314768.s002.docx]

| **Journal requirements** |  |
| --- | --- |
| 1. Please ensure that your manuscript meets PLOS ONE's style requirements, including those for file naming. | We have reformatted the manuscript in line with the style requirements. |
| 2. Thank you for stating in your Funding Statement:  This work was supported by the Economic and Social Research Council: HW is in receipt of PhD funding from the London Interdisciplinary Social Science Doctoral Training Partnership [grant number: ES/P000703/1], <https://liss-dtp.ac.uk>  Please provide an amended statement that declares *all* the funding or sources of support (whether external or internal to your organization) received during this study, as detailed online in our guide for authors at <http://journals.plos.org/plosone/s/submit-now>.  Please also include the statement “There was no additional external funding received for this study.” in your updated Funding Statement.  Please include your amended Funding Statement within your cover letter. We will change the online submission form on your behalf. | Revised statement:  This work was supported by the Economic and Social Research Council: HW is in receipt of PhD funding from the London Interdisciplinary Social Science Doctoral Training Partnership [grant number: ES/P000703/1], <https://liss-dtp.ac.uk>. There was no additional external or internal funding received for this study. |
| 3. When completing the data availability statement of the submission form, you indicated that you will make your data available on acceptance. We strongly recommend all authors decide on a data sharing plan before acceptance, as the process can be lengthy and hold up publication timelines. | This statement is no longer relevant, as we have now uploaded the data to the Open Science Framework (<https://osf.io/6rdvf/?view_only=9b901d9f4f504dd3877dc3f9dbc0848d>). (The OSF link for peer review will be converted to a public page and the link included in the paper upon acceptance.) |
| 4. We note that this data set consists of interview transcripts. Can you please confirm that all participants gave consent for interview transcript to be published? | We can confirm that all participants gave consent for their anonymised interview transcript to be uploaded to an online repository. |
| 5. Please review your reference list to ensure that it is complete and correct. | All references have been checked and we can confirm that the list is complete and correct, so no changes were made. |
| **Editor Comments** |  |
| Please review the references, some with authors names and some without, apply consistent referencing as per journal guide, throughout. | Thank you for highlighting this, we have removed all names. |
| Kindly indicate the impact of the survey prior to the qualitative interviews and address the reasoning behind describing the study as qualitative when a quantitative method was added/utilised. | The survey that was conducted was part of a separate (as yet unpublished) study regarding the predictors of sitting time. The only data used from the survey for the current study related to participant demographics. The present study meets the definition of a ‘qualitative study’ in that only qualitative data are used to address the research question; quantitative data are used only for sample description purposes.  We have added a sentence explaining that the demographic information collected via this survey was used to describe the sample (line 98 in track changes document). We have also expanded our reflections, in the limitations section of the discussion, on the potential impact of completing a survey on the interview data (lines 410-418). |
| I would expect to see something about the long term problems relating to excessive sitting while studying that were mentioned, viz., diabetes mellitus, to name one, in the discussion/conclusion section. It was addressed/raised in the introduction but there was no follow up. Is there a significance to the chronic illnesses that were mentioned as being potentially problematic, and possibly due to a sedentary student life? Please add this in to complete this thread in the article. | Thank you for your comment, we agree this should have been picked up in the discussion. We have added two sentences in the first paragraph of the discussion (line 351 and 356-8) to highlight the point that our participants reported high levels of sitting and, given the adverse physical and mental health outcomes associated with this, interventions are needed to help students reduce their sedentary behaviour and increase their PA. |
| **Reviewer Comments** |  |
| **Reviewer #1:** The authors showed great understanding and knowledge about the subject matter. This study will help researchers and practitioners to understand the subject matter better to help develop proper techniques to help sedentary lifestyles while improving learning | We thank the reviewer for their positive comments. |
| **Reviewer #2:** The sample size of this research, although has a qualitative design it is small. The findings of this study cannot be generalized since there are so few participants. The present paper does not bring new insight to the knowledge of the field. I don't consider the paper suitable for publication in this Journal. | While we note the reviewer’s concerns around sample size, such concerns are less valid to qualitative research. The aim of a qualitative study like ours is to document reflections and perspectives on a topic, not to provide definitive and generalisable conclusions. See for example Smith (2018, p137), who states that “*it is a misunderstanding to claim that qualitative research lacks generalizability […] statistical types of generalizability that inform quantitative research are not applicable to judge the value of qualitative research or claim that it lacks generalizability*”; <https://doi.org/10.1080/2159676X.2017.1393221>). |
